# Misled about lead: an assessment of online public health education material from Australia’s lead mining and smelting towns

**DOI:** 10.1186/s12940-015-0085-9

**Published:** 2016-01-06

**Authors:** Marianne Sullivan, Donna Green

**Affiliations:** Department of Public Health, William Paterson University, Wayne, NJ USA; Climate Change Research Centre, University of New South Wales, Sydney, NSW 2052 Australia; The ARC Centre of Excellence for Climate System Science, University of New South Wales, Sydney, NSW 2052 Australia

**Keywords:** Blood lead levels, Broken Hill, Mount Isa, Port Pirie, Lead smelting, Childhood lead exposure

## Abstract

**Background:**

This study assesses the accuracy and comprehensiveness of online public health education materials from the three Australian cities with active lead mines and or smelters: Broken Hill, Mount Isa and Port Pirie.

**Methods:**

Qualitative content analysis of online Australian material with comparison to international best practice where possible.

**Results:**

All materials provided incomplete information about the health effects of lead and pathways of exposure compared to best practice materials. Inconsistent strategies to reduce exposure to lead were identified among the Australian cities, and some evidence-based best practices were not included. The materials normalised environmental lead and neglected to identify that there is no safe level of lead, or that primary prevention is the best strategy for protecting children’s health.

**Conclusions:**

Health education materials need to clearly state health risks from lead across developmental stages and for sensitive populations, integrate a primary prevention perspective, and provide comprehensive evidence-based recommendations for reducing lead exposure in and around the home. Families who rely on information provided by these online public education materials are likely to be inadequately informed about the importance of protecting their children from exposure to lead and strategies for doing so.

## Background

### Australia’s lead mines and smelters

After China, Australia is the world’s largest primary lead producer, with the world’s largest lead smelter located in Port Pirie, South Australia [1]. This smelter, built in 1889, processed ore brought by train from the Broken Hill lead mine, NSW - a mine that is still operational today. The city of Mount Isa, Queensland, also has an operational mine and a lead and copper smelter. Each of these three cities has between 13,000 and 23,000 people living in the immediate vicinity of the mining and/or smelting operations. In addition to these sites, there are also smaller active lead mines in Tasmania and the Northern Territory.

Over the last century, these regional towns have developed a significant reliance on the lead industry for their economic base [2]. Concerns have been raised in recent years that industrial capture of these cities, and their respective state governments, has occurred in relation to how pollution monitoring and regulation is being managed; [3] similar to processes that have occurred internationally [4]. In several instances, concerns have been raised that corporate profit is being prioritised over public health, specifically in relation to the use of pollution control technologies and the awareness of the possibilities of reducing the impacts of lead on health [5].

### Lead and human health

Lead exposure adversely affects cardiovascular, immune, endocrine, and reproductive systems. In children, even at BLLs < 5 μg/dL, lead exposure is linked to attention-related behaviour problems, IQ decrements, poorer academic performance; and in older children, possibly leads to puberty delays and renal effects. Adults are also vulnerable to the effects of low-level lead exposure, with an increased risk of renal effects at BLLs < 5 u μg/dL, adverse cardiovascular outcomes at BLLs <10 μg/dL, and adverse effects during pregnancy at BLLs <10 μg/dL [6]. In 1993, the National Health and Medical Research Council (NHMRC) recommended that Australians have BLLs of 10 μg/dL or lower. In 2015, this level was revised to 5 μg/dL [7]. This reduction is consistent with action taken by the US Centers for Disease Control (CDC) in 2012 [8].

### Blood lead levels in leaded cities

Since the earliest operations of the Australian mines and smelters over a century ago, occupational lead poisoning has been a significant public health concern [9]. In the 1980s and 1990s, widespread exposure to lead among children living in these cities was documented [10–12]. In Broken Hill, the most recent data show that 53 % of children tested have BLLs over 5 μg/dL, with Aboriginal children “twice as likely to have blood lead levels that exceed 10 μg/dL” [13]. In Mount Isa the scope of the problem is more difficult to assess since the most recent published children’s blood lead level (BLL) data are from 2010. Out of a small sample of 167 children, approximately five per cent had BLLs of 10 μg/dL or higher, and Indigenous children had a statistically significantly higher geometric mean blood lead level compared to non-Indigenous children (5.44 compared to 3.98 μg/dL) [14]. In 2008, a larger sample showed a greater percentage of children with BLLs greater than or equal to 10 μg/dL (11.3 %) [15]. In Port Pirie, in the first half of 2014 approximately 20 % of children tested had blood lead levels of 10 μg/dL or higher [16]. Taylor et al. report that in Broken Hill and Port Pirie, average BLLs appear to have risen in recent years [15].

### Lead education materials

In Broken Hill, Mount Isa and Port Pirie, there are lead health education programs which aim to educate the respective communities about lead health risks and strategies for preventing exposure. In each city, lead health education materials have been developed. These materials are disseminated online, via printed flyers and by personal interactions at local awareness raising days. Table 1 identifies the three programs, the agencies and companies involved in each and compares key aspects of their online materials, including the nature of their online presence, currency of website news, and details subsections and links to other resources. All of the programs are described as a partnership of local and state government agencies. In Mount Isa and Port Pirie the mining companies are also identified as participants. Each program has provides similar materials (for example FAQ, subsections on pregnancy, renovating etc), however, there are differences between their wider social communication strategy, for example via facebook, twitter, or other online newsletter options. None of the sites have ‘current’ news associated with the website, (with currency judged as an update provided within the last 3 months).

**Table 1:** Comparison of online material provided by each location

	Broken Hill	Mount Isa	Port Pirie
Program Title	Lead Health Program	Living with Lead Alliance	Targeted Lead Abatement Program; Ten for Them
Slogan	“Lead it’s in our hands”	“Living safely with lead”	“It’s in our hands”
Partner Organizations	The Child & Family Centre, Broken Hill Health Service; NSW Government, Far West Local Health District	Queensland Government (Health and Department of Environment & Heritage Protection); Mount Isa Mines; Mount Isa City Council	South Australian Government; Nyrstar
Online FAQ	No	yes, although only on blood lead level	yes, comprehensive list
Online newsletter	No	Yes	No
Photo or video gallery	No	No	Yes
Links to other resources	no active links on page	No	yes, with broken links
Clearly identified subsections on:			
• Living with lead	No	Living with lead poster	Yes
• Renovating	Yes	Lead in the home poster	Yes
• Garden	Yes	Lead in the back yard poster	Yes
• Expectant mothers	Yes	Pregnancy poster	Yes
• Nutrition	Yes	Diet poster	No
• Hygiene	Yes	Wet wipe poster	No
• Occupation	Yes	No	No
Other online resources	Schematic of lead testing schedule	multiple posters	Top tip cards, community van
Currency of news section	~2014	Nov 2013	March 2015
Facebook	No	Yes	Yes
Twitter	No	yes - although with only 9 tweets	yes, but broken link

The projects that produce these public health materials all state similar objectives which include the minimisation of the impact of lead exposure whilst allowing the lead industry to continue to operate. Specifically, the *Broken Hill Lead Health Program* states their objective is “minimising the impact of lead exposure whilst maintaining a viable mining industry in Broken Hill” [17]. The *Living with Lead Alliance* was established “to develop and deliver an extensive and ongoing public education campaign to ensure the health of Mount Isa residents.” The Alliance’s campaign, entitled ‘Living Safely with Lead’ is designed to “remind us that Mount Isa is a safe place to live” [18]. The *Ten for Them/Targeted Lead Abatement* campaign, aims to “ensure that children between 0 and 4 years of age, living in Port Pirie, have blood lead levels as low as possible” [19].

### State health department health education materials

In addition to the city specific online material, each State’s public health department has online health education materials specific to lead exposure. There is variation in the content of this educational material for each state with only South Australia Health providing detailed and specific information about risks to children living in a leaded city. A review of state health department materials also indicates that there is no inter-state coordination on lead advisory or BLL. For example, on 17^th^ July 2014, Queensland Health announced that it would adopt new blood lead notification levels of 5 μg/dL in response to the (then) draft NHMRC report [20]. South Australia Health has similarly indicated its support of the new blood lead notification levels and states that these will be used specifically in Port Pirie [21]. New South Wales Health however, does not mention the NHMRC’s new blood lead notification level on their website.

Additionally, South Australia Health has multiple web pages that address lead health risks. A page with Frequently Asked Questions directly addresses lead exposures in Port Pirie and provides information on testing and reporting Port Pirie children’s blood lead levels [22]. This health department also has a general page on lead exposure and domestic reduction actions that can be undertaken [23]. SA Health also maintains a fact sheet titled “Lead and your health” which mentions an increased risk of exposure in mining and smelting communities [24].

In contrast, Health Queensland does not provide information on health risks to children living in Mount Isa. The Department has a general one page brief on lead in house paint, but for further information, links to the national standards and information provided by the NHMRC [25]. Other online materials relate to worker health and safety but do not indicate any geographically specific locations of particular concern [26].

NSW Health has an online fact sheet relating to lead exposure in children but this fact sheet does not mention lead exposures in Broken Hill and does not go into detail about mining community exposures [27]. There are no other identifiable online fact sheets aimed at the public available from NSW Health that relate to lead exposure or lead health risks.

### Efficacy of lead health education

The inferred premise of lead health education in mining/smelting cities is that increased knowledge about the health risks of lead, the pathways of exposure, and strategies for preventing exposure will spur behaviour changes that will reduce exposure, and ultimately result in lowered blood lead levels. However, the efficacy of lead health education in lowering children’s blood lead levels has not been conclusively demonstrated. A recent review found that household educational and dust control interventions appeared to be ineffective at reducing children’s BLLs [28]. One of the studies included in the meta-analysis was conducted in Broken Hill and included home and yard remediation in addition to public health education, but reported limited effects on children’s BLLs [29].

Lead health education efforts are complicated by the fact that young children who have the highest risk of exposure to lead due in part to developmentally appropriate behaviours such as playing outside in dirt, hand to mouth activity, and crawling on floors, are not able to understand the risks posed by a lead contaminated environment. Lead health education, therefore, is typically aimed at parents and caregivers for the purpose of educating them about their children’s risk of exposure, the adverse health effects of lead, the behaviours and activities that increase risk, and actions parents can take to lower children’s risk.

## Methods

We used qualitative content analysis to evaluate the accuracy and comprehensiveness of online lead health education information provided to residents of the three cities. Our primary interest in this study was to elucidate ‘manifest content’, that is, to develop codes and categories that describe the actual content of education materials [30]. Due to the updated NHMRC advisory level for blood lead levels in May 2015, we re-checked the materials 2 months after this date to ensure any updates made to material would be captured in our assessment. We included information in the text and graphics from all of the direct webpages linked to each of each site, but for consistency, did not follow links that took the viewer off the main website.

Initially we determined the unit of analysis; [31] the online lead health education materials aimed at parents produced by Broken Hill Child and Family Health Centre, identified online as: *Lead it’s in our hands*; [17] Mount Isa’s Living with Lead Alliance, identified online as: *Living safely with lead*; [18] and Port Pirie’s *Targeted lead abatement program* [19].

We then used an open coding process to comprehensively identify categories and concepts that emerged from the data. From an initial list of open codes, we determined higher-order categorisations and groupings [31]. Based on our initial open coding, we identified three over-arching domains in which the content of the health education materials clustered: health effects of lead, exposure pathways, and strategies for reducing exposure. Because our primary objective was to assess the accuracy and completeness of the lead health education materials, we determined the best way to do this would be to compare the materials from the mining and/or smelting cities to best practice materials. We defined best practice health education materials as those based on current scientific evidence of health effects (e.g. no safe level of lead exposure identified in children, health effects of low-level lead, identification of vulnerable populations, and delineation of health effects across developmental stages), and those that most comprehensively addressed sources and pathways of exposure. Materials considered included those produced by the World Health Organization, Australian national and state governments, and US national and state governments. In addition the materials had to be in the English language, intended for parents/consumers rather than providers, and web-based.

For the first domain, the health effects of lead, we determined that the best practice materials were those developed by the CDC, due to their comprehensive consideration of lead’s health effects across developmental stages, consistency with recent scientific evidence, and focus on parent/caregiver education [32]. Consequently, we developed codes to capture the content of the CDC materials through open coding of the materials and then organised these codes into higher order categories.

For the second domain, pathways of exposure, we determined that best practice lead health education materials were those developed by the NHMRC [33], due to their comprehensive discussion of exposure pathways and their specificity to the Australian context. WHO [34] and CDC materials were ruled out as “best practice” because they provided only short descriptions of sources and pathways of exposure and did not go into detail about the mining/smelting context. Additionally, the NHMRC materials specifically address some pathways particular to Australia (e.g. rainwater collection for household use). Codes for this domain were developed in the same process described above for the first domain.

For the third domain, strategies for reducing exposure, we could not identify best practice materials specific to the mining/smelting context, therefore, we made the decision to develop an exhaustive list of codes, organised into categories, that would capture the advice provided to parents across the cities being assessed. This enabled us to to analyse the recommendations provided in each city, to compare each city to the others, and where possible, to compare the recommended strategies to the published literature.

Once the codes in each of the three domains were finalised the authors independently coded the health education materials. Then the codes applied by the authors were compared, and any areas of disagreement were discussed and reconciled.

## Results

### Domain 1. Health effects of lead

In comparison to the CDC health education materials that state, “No safe blood lead level in children has been identified” [35] none of the materials made a comparable unequivocal statement regarding BLLs in children. While the CDC materials state that the “effects of lead exposure cannot be corrected” [35] only the Broken Hill materials addressed the fact that permanent damage may result from lead exposure noting “irreversible intelligence loss” is possible [36]. The CDC materials assert that while “even low levels of lead in blood” affect children [35], only the Broken Hill materials identify “low levels” of lead as being of concern [36]. Both the Mount Isa and Port Pirie materials point to 10 μg/dL as the BLL at which children may begin to experience health effects. These materials are out of date as the NHMRC guidelines were reduced to BLL of 5 μg/dL 2 months prior to the analysis [37, 38].

The CDC educational materials stress the importance of primary prevention, that is “prevent [ing] lead exposure to children before they are harmed” [35] (emphasis in original). This primary prevention message is absent from all the materials examined.

With respect to health effects on children, the CDC materials note lead’s pervasive impacts, stating “lead exposure can affect nearly every system in the body” [32]. In comparison, the Broken Hill material points to lead’s ability to damage the circulatory system, kidneys, reproductive system, and brain [36]. The Mount Isa and Port Pirie materials discuss health effects in the context of BLLs >10 μg/dL, and note that at these levels lead can affect the “development of organ systems” (Mount Isa) [37], and “development of internal organs” (Port Pirie) [38, 39], specifically the brain and the central nervous system. None of the assessed health education materials stress that lead can affect most systems of the body.

The CDC materials discuss the developmental effects of lead, noting that low blood lead levels “have been shown to affect IQ, ability to pay attention, and academic achievement” [35]. Broken Hill provides the most accurate and comprehensive information on developmental effects of the three leaded cities, pointing to “irreversible intelligence loss, learning difficulties, behavioural problems and hyperactivity” [36]. The Mount Isa materials specify impacts on “neurobehavioral function” and note that lead is linked to IQ loss, but imply that this occurs at 10 μg/dL or higher stating: “1–3 IQ points per 10 μg /dL increment in population blood lead levels” [37]. The Port Pirie materials are even less specific, and point to effects on “intellectual performance and general behavior” [38].

While all of the materials note that lead is transferred during pregnancy from a woman to her fetus, specific information regarding the link between lead and adverse fetal outcomes is lacking in all three leaded cities. In contrast to the CDC materials, none of the educational materials note that lead exposure during pregnancy can “cause your baby to be born too early or too small” [40]. Contrary to the CDC materials, none of the assessed material identifies that lead exposure during pregnancy can increase the risk of miscarriage [40].

Where CDC provides specific fetal/infant organ systems that may be affected by lead if the mother is exposed, (e.g. “too much lead in your body can hurt your baby’s brain, kidneys and nervous system”) [40] the Broken Hill and Mount Isa materials note that the fetus is at risk, but do not state specific risks [41, 42]. In the Port Pirie materials, no specific fetal health risks are discussed.

All of the materials identify young children as being at increased risk due to biological factors such as rapid growth and development, and behavioral factors such as hand-to-mouth behavior. However, inconsistencies were identified in the age of children said to be most at risk. In the CDC materials children under six are said to be at highest risk [35], while in Broken Hill and Port Pirie this is stated as children under five [39, 41]. The Mount Isa materials point to children under four as being at highest risk of negative health effects from lead [37, 42].

Specific information on BLLs at which public health intervention should begin, while highlighted in the CDC materials [32], are absent from materials for Broken Hill and Port Pirie. For Mount Isa, the materials note that if child has BLL > 10 μg/dL, Queensland Health “may sample and analyse soil, dust and paint flakes…” but do not communicate that there is a BLL at which intervention is recommended [43]. Additionally, information on the BLL at which medical treatment should occur while present in the CDC materials [44] is absent in the assessed materials. Only the CDC [32] and Broken Hill materials specifically note that lead exposure is often asymptomatic, and only the Broken Hill materials provide a specific recommendation on when children should first get a blood lead test (12 months) and when they should be tested thereafter (18 months, 2, 3 and 4 years of age) [36].

Finally, CDC notes disparities in lead exposure by race/ethnicity and income [45]. Only the Broken Hill materials discuss disparities in leaded cities, reporting that average BLLs are higher among Aboriginal children [46].

### Domain 2. Pathways of lead exposure in mining and smelting cities

The pathways of lead exposure discussed in the assessed material were evaluated in comparison to the online NHMRC FAQs material [33], which primarily discusses how people may be exposed to lead. These FAQs include information specific to mining/smelting areas and identifies them as places where people “can be exposed to higher levels of lead than are found in other areas” [33].

### Source

The NHMRC FAQs identify the source of lead contamination in mining and smelting areas as originating from industry. All of the materials from the three leaded cities acknowledge that a source is the local mining/smelting industry as well as natural contamination. For example, in addition to “mining and smelting activities for over 100 years,” the Broken Hill materials point to “considerable natural contamination of soils,” [36] and the Mount Isa materials state “lead is present in the region naturally and from industrial activities” [47].

### Transport

With respect to how lead moves from the source into residential areas, the NHMRC FAQs attribute contamination to “air pollution that contains lead which contaminates the local dust and soil” [33]. In the Broken Hill materials no explanation for how lead gets into homes and yards is provided. The Mount Isa materials state that “a significant source of lead can come from dust that enters the home environment via prevailing winds that blow dust containing lead from the smelter” [37]. The Port Pirie materials are the most informative, even identifying the part of the city that is most at risk: “The strong north/north-west winds blow across the smelter, picking up emissions and lead dust on the way, then deposit them predominantly in the south and south-east areas of the city” [38].

### Route of exposure

The NHMRC FAQs identify ingestion as the way lead enters children’s bodies as children “put things in their mouths, touch dusty surfaces indoors and outdoors, and touch their mouths more” [33]. While the materials from the three cities all point to ingestion as the primary route by which lead enters children’s bodies, the Mount Isa materials provide the clearest explanation of how this occurs: “young children are more likely to play in dirt and to place lead contaminated hands and other objects in their mouths.” [37] These materials also note that lead dust “can be found on children’s hands at all ages” [37]. The Broken Hill materials state “the normal exploratory hand-to-mouth activity of children increases their risk of ingesting lead” but do not make concrete the specific behaviors and activities of children that increase their risk [41]. The Port Pirie materials explain children’s ingestion similarly [39].

Although inhalation is mentioned as a way that lead can enter the body, only the Port Pirie materials specifically discuss inhalation of fine particulate matter from mining and/or smelting emissions as a route of exposure. The NHMRC materials also do not mention inhalation exposure specific to mining/smelting areas. The Mount Isa materials note that children can breathe in lead dust when lead paint is sanded [37], but do not mention that fine lead particles that are emitted from smelters may be inhaled [5, 48–50] and that particles that settle in the environment can be re-suspended by wind [51], or inside the home by sweeping or vacuuming, and thereby inhaled [52].

### Contaminated media

Consistent with the NHMRC FAQs, all of the materials discuss contamination of local dust and soil with lead. The Broken Hill materials are most specific, noting that “many local yards exceed the national soil lead safety level” and that “bare soil and dust is the biggest source of lead and risk to children and pets” [53]. In the Port Pirie materials, a more general statement is made: “It can be assumed that all soils in Port Pirie contain some level of contamination” [54]. The least comprehensive discussion is found in the Mount Isa materials which note “Parts of the city have natural mineralisation with lead and other metal ores and traces of historic mine sediment” [47]. None of the materials contain information on the acceptable Australian standard for lead in residential yards (300 mg/kg) [55], or the percentage of yards that exceed the standard.

The NHMRC FAQs and the health education materials from all cities note that rainwater may be contaminated with lead, but only the Broken Hill materials specify how this can occur, through “dust on the roof, old pipes, old tanks and leaf matter” and further, that “levels of lead in rainwater tanks can change on a daily basis and is generally high” [56].

### Take-home exposure

All of the health education materials note that workers traveling from the mining/smelting sites can transport lead into the home. The NHMRC FAQs provide the most comprehensive information on take-home exposure, noting that lead can travel home with workers not only on their bodies and clothes, but also on personal possessions such as cell phones. NHMRC materials also note that cars can transport lead to the home. The Broken Hill materials mention take-home lead on clothes but not on possessions or vehicles [57]. The Port Pirie materials mention “bodies, clothing and possessions” and also mention specific common possessions like cell phones and glasses [39, 58]. Both the Port Pirie and Mount Isa materials note that vehicles leaving the site can transport lead into the community [58, 59].

### Domain 3. Strategies for reducing exposure

The health education materials provide extensive recommendations for actions that parents or residents can take to reduce lead exposure, particularly for children. Across the three cities, we identified over 150 suggested actions. They fall in categories related to: exterior and interior cleaning, garden, hygiene, diet and food preparation, children’s toys, rainwater, pets, and laundry. Because we could find no other comprehensive source of exposure reduction strategies to use for comparison purposes, we describe the recommendations made, comparing the content among the three cities, and reporting where major recommendations are either consistent or inconsistent with recommendations made by government agencies in Australia and/or the US.

### Tracked in lead

With respect to cleaning the threshold of the home to reduce tracked in lead, only the Mount Isa materials make specific recommendations to use a door mat, to wash the mat, and to clean doorsteps, porches, verandahs and entryways [37, 47]. Tracked in dust and dirt has been identified as a source of interior lead [60], and the US EPA recommends the use of doormats both outside and inside to reduce tracked in lead [61]. No recommendations for exterior or threshold cleaning or use of door mats are made in the Broken Hill or Port Pirie materials.

All of the materials contain recommendations to remove shoes when entering the home, consistent with CDC recommendations [35]. However, the recommendations in the leaded cities are often not as clear, and are inconsistently presented. In the Broken Hill materials “dusty shoes” are to be kept outside [56]. But elsewhere residents are told that after working in the garden they can either “wipe shoes or leave outside” [53]. In the Mount Isa materials “yard shoes” are to be left outside and work boots are to be kept in a “storage box” outside [47]. Elsewhere residents are told to “leave shoes outside to reduce the amount of soil you bring into your home” [62]. In the Port Pirie materials parents are told “it is certainly a good idea to always leave their shoes outside” if they “have young children who sometimes play on the floor” [38]; elsewhere they are told to leave “your shoes” outside and elsewhere “leave your kids’ shoes outside” [63]. None of the materials provide a consistent message.

### Interior cleaning

All of the materials address the importance of cleaning inside the home, and all note the need to use damp or wet cleaning methods. However, only the Mount Isa materials provide an explanation for why wet cleaning needs to be carried out [59]. Neither the Broken Hill nor the Port Pirie materials explain the importance of minimising the release of lead dust into household air while cleaning. Significantly, only the Port Pirie materials mention the use of a HEPA filter equipped vacuum, and they state only that such filters are “preferred” [38]. The advice in the US to use vacuums with HEPA filters for cleaning up after renovations involving lead paint is long-standing [64]. Further, the US EPA recommends providing HEPA filter equipped vacuums to residents in lead contaminated Superfund sites, including mining and smelting communities [65]. HEPA filter-equipped vacuums are also recommended by South Australia Health for people living in “lead contaminated environments (e.g. near smelters)” [66]. While all materials suggest removing children from the room while vacuuming, only the Port Pirie materials explain that vacuuming can increase exposure. Neither the Broken Hill or Mount Isa materials explain that that vacuuming has been shown to re-suspend dust into the air that can be inhaled or ingested [52].

Because lead accumulates in carpet, it is another potential source of domestic exposure to lead for children [67, 68]. In Broken Hill, residents are advised to “remove old carpets,” but are not provided with specific advice on how to do this safely [56]. US EPA recommends that if lead contaminated carpet is to be removed, that a series of steps are followed to minimise the spread of lead dust [67]. In the Port Pirie materials, the lead health education materials state that “carpet is always a good choice, but the most important thing is that you keep existing floor coverings clean, no matter what they are” [38]. The Mount Isa materials do not give recommendations on carpeting or other floor coverings.

Other directives related to interior cleaning lack specificity—in the Broken Hill materials, parents are told to “wash, blankets, bedding and toys” but are not told how often they should do this. Similarly they are told to wet mop “regularly” [56]. Only in the Mount Isa materials are residents provided with specific recommendations on how often wet mopping should occur “at least once a week,” and Mount Isa residents are also told to “regularly” clean their mops [59]. The once per week recommendation is consistent with US EPA recommendations on mopping and cleaning window sills in homes with deteriorating lead paint [61, 67].

### Garden

In the educational materials, the garden is noted as a place where children may be exposed to lead contaminated soil. However, recommendations for managing this risk vary. Only in the Broken Hill materials are residents specifically told that soil contamination beyond Australian standards is widespread and that “bare soil and dust is the biggest source of lead and risk to children” [53]. None of the educational materials notify parents that the Australian standard for lead in residential soil where there are accessible gardens is 300 mg/kg [55]. Residents in all three communities are told to cover bare soil.

In reference to growing vegetables at home, the Mount Isa and Port Pirie materials mention using clean soil and raised beds [54, 69] whereas in Broken Hill residents are advised less specifically to “build up garden beds with compost, mulch and clean soil especially those intended for growing fruit and vegetables” [53]. US EPA recommends replacing contaminated soil with 24 in. of clean soil for gardens in lead contaminated areas [65], and a recent US EPA Technical Review Workgroup report recommends not allowing children to garden if soil metal concentrations have not been measured [70].

### Children’s play areas

There is also different advice on maintaining children’s play areas. In Broken Hill, parents are advised to cover bare soil or “fence off a grassed play area if your yard is too big to maintain” and to provide children with a sandpit, covered when not in use, filled with “white beach sand” that does not mix with the soil [53]. The Mount Isa materials recommend using “clean topsoil and healthy turf or groundcover” for children’s play areas [47, 62], and sandpits are also recommended, though the advice provided on siting them is not as extensive as in Broken Hill. No specific recommendations for children’s play areas are made in the Port Pirie materials beyond general advice to cover bare soil. There is no specific advice to provide a sand pit for children in the Port Pirie materials, though if residents have one, they are advised to cover it [38]. Finally, only the Port Pirie materials advise washing outdoor play equipment [54]. Dust on outdoor play structures has been shown to be a source of lead exposure to children, and washing playground equipment has been found to be of limited efficacy in the context of on-going deposition [71, 72].

### Hygiene

Hygiene is a topic that receives a great deal of attention, and hygiene recommendations are directed at both children and adults. All recommend regular handwashing, for example, before eating, after gardening, after outdoor play. This is consistent with US EPA recommendations [67]. In Mount Isa and Port Pirie, drying hands is also emphasised [63, 69]. In the Broken Hill and Mount Isa materials, parents are advised to keep fingernails short and to use a nail brush, however, advice on children’s nails is not provided in Port Pirie. In the Broken Hill materials parents are advised to “discourage dirt eating, sucking fingers/toys” [56]. In the Mount Isa and Port Pirie materials parents are advised to “wash and dry hands” before touching or playing with their children [37, 63].

### Dietary advice

Significant attention is given to dietary advice. Parents in all three cities are advised that children should not have “empty stomachs” and should follow specific diets in order to, according to the Broken Hill materials, “keep children’s lead levels low” [73]. All three specify that children should have diets that include iron, vitamin C, and calcium to help prevent lead absorption, which is consistent with the CDC Advisory Committee’s statement on low-level lead poisoning [74] and advice provided by South Australia Health [75]. The leaded cities’ materials also recommend limiting or avoiding fatty foods [63, 73, 76]. Mount Isa materials go the furthest with specific dietary recommendations and advise that children should also consume foods with zinc, magnesium, and garlic and protein [76]. According to the CDC’s Advisory Committee on Childhood Lead Poisoning Prevention, “evidence that nutritional interventions affect BLLs is limited. However, higher dietary calcium, iron, vitamin C, and zinc have been associated with lower blood lead concentrations at least in infancy” [74].

### Home-grown food

Only in Port Pirie are there recommendations for avoiding food potentially contaminated with lead from home gardens where children and pregnant women are advised not to eat “leafy vegetables, like lettuce, silverbeet, cabbage, broccoli and cauliflower” due to the difficulty of removing lead particles [54]. This is not mentioned in any of the other health education materials. In the Broken Hill materials, “homegrown produce” is said to be “fine as long as it is grown in fresh soil and washed well” [56]. A Technical Review Workgroup (TRW) for US EPA recently recommended that not consuming outer leaves of leafy vegetables if soil lead concentrations exceed 100 ppm [70].

Divergent advice is given on food preparation. All materials advise residents to wash fruit and vegetables before eating, however, in the Port Pirie materials, residents are told in addition, to “leave skins on fruit and vegetables whenever possible” [63] for nutritional reasons, but in the Mount Isa materials, residents are told to “always…peel root vegetables before eating” [69]. The US EPA TRW report on garden soils recommends peeling home-grown root vegetables if soil concentrations of lead are over 100 ppm [70].

### Rainwater

While all materials address lead in rainwater, the most comprehensive advice is given in the Port Pirie where residents are advised that rainwater should not be used for drinking, cooking, making “baby formula, baby foods and/or the sterilisation of baby equipment,” and that boiling rainwater will further concentrate lead [38]. In the Mount Isa materials, residents are told that rainwater can be “an exposure source” and that boiling water will “not remove lead”, but no specific advice not to drink it or use it for other purposes is given [37]. In the Broken Hill materials residents are told that they should not drink rainwater but are advised to use it on the garden without specifying whether they should avoid watering their vegetable gardens with this water [56].

### Pregnancy/infants

Specific advice is given to pregnant women in Broken Hill and Mount Isa for the purposes of reducing lead exposure during pregnancy. In the Broken Hill materials, women are told that when they are “planning or starting” their families is “the time to start planning for lead.” They are advised that following a “healthy diet” (including iron, calcium, zinc, Vit C, low-fat) “will help lower leads [sic]” [77]. CDC’s Prevention tips for pregnant women recommend “eating foods with calcium, iron and vitamin C” but they state the possible benefits with less certainty: “these foods may help protect you and your unborn baby” [40]. In the Mount Isa materials, pregnant women are given similar dietary advice [78], and in both Broken Hill and Mount Isa, pregnant women are informed of specific places/activities where they may be exposed to lead [77, 78]. There is no such advice provided to pregnant women in the Port Pirie materials, in fact expectant mothers are told: “protecting unborn children and giving them the best start in life is easy: just have your own blood tested during pregnancy” [63]. All of the materials specify that pregnant women should not be present during household renovations [79–81].

The Port Pirie materials have the most extensive recommendations for preparing the house and the baby’s room before birth in order to decrease lead exposure. Expectant mothers are also offered the opportunity to consult with the South Australian government’s Port Pirie Environmental Health Centre [82]. No similar advice on preparing for birth is given in the Broken Hill or Mount Isa materials. The Broken Hill [56] and Mount Isa materials both mention that dummies should be kept clean, and in the Mount Isa materials further advice is provided to keep dummies “pinned to clothing” [69]. Only in Broken Hill are parents told to keep baby bottles clean, though the bottle nipple is not specifically mentioned [56].

### Pets

Parents are told in all three cities to wash children’s hands after petting animals [56, 62, 63]. The Broken Hill and Mount Isa materials recommend that pets, particularly dogs, be kept outside [56, 62]. Additionally, pets are to be kept clean/washed regularly in Broken Hill and Mount Isa [56, 62].

### Laundry

Only in the Port Pirie materials is the practice of hanging laundry outside to dry addressed. Residents are cautioned that laundry hanging outside can collect lead dust. They are advised not to hang out their wash on windy days or overnight [38].

## Discussion

While the effectiveness of lead health education for reducing children’s BLLs has not been demonstrated [28], public health authorities have a duty to communicate the risks of lead exposure in mining/smelting communities and to advise parents of steps they can take that may possibly reduce risks. Health educators, however, should be cognisant that health education aimed at reducing exposure to lead in household dust and residential yards by itself has been insufficient to reduce children’s BLLs to 5 μg /dL or lower to date.

Health education/promotion theory provides a framework for the development of health education materials. Often the Health Belief Model (HBM) is applied to developing educational materials that encourage people to change their behavior in order to avoid or mitigate health risks. The HBM specifies in part that before people will take action to change behavior they must “believe they are susceptible to the condition,” that the condition “has serious consequences,” and that by taking action they can “reduce risks” [83]. Therefore, lead health education materials must fully and accurately lay out the risks of lead exposure for different populations (pregnant women, infants, children and adults) so that residents will understand the importance of taking the extensive actions that are recommended to reduce risk. Additionally, risk communication frameworks are also useful to consider here. Current thinking in the field of risk communication is to move away from expert driven technical communication of risk toward cultural approaches to risk communication that value community engagement, local knowledge of risk and exposure, and stressing openness and transparency [84, 85].

Based on our analysis, the health education materials from all three Australian mining and smelting cities fail to provide consistent, accurate and comprehensive information on lead health risks that would be the underpinning for people taking such extensive actions to protect their children from lead, or themselves, in the case of pregnant women. While the Broken Hill materials were the most accurate and comprehensive from the standpoint of communicating the health effects of lead, all three are missing critical information with respect to lead’s health effects. Additionally the materials understate internationally accepted health effects of lead exposure, particularly during pregnancy, and for fetal growth and development. Additionally, the Mount Isa and Port Pirie materials fail to communicate that the harm from lead is permanent and irreversible. All three lack a critical emphasis on primary prevention—preventing exposure before it occurs--as well as clearly communicating that “protecting children from exposure to lead is important to lifelong good health” [35]. The Mount Isa materials contain information that is erroneous, specifically: “the World Health Organisation, Centre for Disease Control, American Academy of Paediatrics and the Australian guidelines indicate that an elevated blood lead level in children is a reading of >10mcg/dL” [42].

The materials do not present a clear picture of the pathways of exposure to lead in mining and smelting communities—information that could be helpful to parents in thinking about ways to reduce risks. The Broken Hill materials in particular could benefit from a clearer statement on how lead is transferred from mining sources into and onto children’s environments. All could improve their descriptions of how children are exposed during different activities, such as playing in the garden, in the home, or at school or daycare. None of the materials recommend practical actions that parents could take such having their soil or house dust tested for lead. Such testing programs are available elsewhere in the country (see, for example, Macquarie University’s “Vegesafe” web page [86]).

Despite the numerous strategies for reducing lead exposure provided to parents, we identified instances in which best practices were not being recommended (e.g. door-mats, HEPA equipped vacuums, recommendations on gardening in lead contaminated soil), as well as inconsistencies across the cities in the advice provided (e.g. peeling vegetables, eating leafy greens from gardens, children’s play areas, use of or cleaning of carpets, among others). Additionally, in some cases recommendations lacked specificity that would likely be helpful to residents, such as whether to wipe or remove shoes, and how often to clean interior spaces. Ideally recommendations on how often to clean interior and exterior spaces would be determined based on site specific data taking into account deposition rates.

In all three communities, the lead health education materials provided little information targeted specifically to the Aboriginal community, which has been shown to be at higher risk of elevated BLLs in Broken Hill and Mount Isa. While the Mount Isa Living with Lead Alliance has produced a brochure entitled “Lead and Your Mob” [87] which pictures Aboriginal parents and children, it does not acknowledge the higher the average BLLs among Aboriginal children.

## Conclusions

Both Broken Hill and Port Pirie have the same slogan for their educational campaign—“Lead—It’s In Our Hands.” Because the materials are aimed at the residents, the slogan implies that residents are able to control children’s exposure through individual actions, though this has not been demonstrated through research [28]. For Mount Isa, the slogan is “Living Safely with Lead.” This slogan is at odds with a primary prevention approach that has been endorsed by leading health bodies –and would include remediating existing lead in the environment and eliminating further environmental contamination [8, 88]. Because lead health education appears to be the main intervention for reducing childhood lead exposure in the three communities we urge that these programs be rigorously and independently evaluated to determine their efficacy.

Families who rely on information provided by these online public education materials are likely to be inadequately informed about the importance of protecting their children from exposure to lead and strategies for doing so. These health education materials need revision to state clearly and consistently health risks associated with lead exposure across developmental stages and for sensitive populations, integrate a primary prevention perspective, and provide comprehensive evidence based recommendations for reducing lead exposure in and around the home. A national, harmonised approach to lead health education led by NHMRC, with community participation, attention to disparities among Aboriginal communities, and based on national and international best practices is recommended.
